# Rocuronium versus saline for effective facemask ventilation during anesthesia induction: a double-blinded randomized placebo-controlled trial

**DOI:** 10.1186/s12871-022-01717-2

**Published:** 2022-06-03

**Authors:** Akira Ide, Natsuko Nozaki-Taguchi, Shin Sato, Kei Saito, Yasunori Sato, Shiroh Isono

**Affiliations:** 1 Department of Anesthesiology, Graduate School of Medicine, Chiba University, 1-8-1 Inohana, Chuo-Ku, Chiba, 260-8670 Japan; 2 Department of Anesthesiology, Chiba University Hospital, Chiba, Japan; 3 Department of Preventive Medicine and Public Health, Keio School of Medicine, Tokyo, Japan

**Keywords:** Anesthesia induction, Mask ventilation, Tidal volume, Neuromuscular blockade, Rocuronium, Obstructive sleep apnea

## Abstract

**Background:**

Mask ventilation progressively improves after loss of consciousness during anesthesia induction possibly due to progression of muscle paralysis. This double-blinded randomized placebo-controlled study aimed to test a hypothesis that muscle paralysis improves mask ventilation during anesthesia induction.

**Methods:**

Forty-four adults patients including moderate to severe obstructive sleep apnea undergoing scheduled surgeries under elective general anesthesia participated in this study. Randomly-determined test drug either rocuronium or saline was blinded to the patient and anesthesia provider. One-handed mask ventilation with an anesthesia ventilator providing a constant driving pressure and respiratory rate (15 breaths per minute) was performed during anesthesia induction, and changes of capnogram waveform and tidal volume were assessed for one minute. The needed breaths for achieving plateaued-capnogram (primary variable) within 15 consecutive breaths were compared between the test drugs.

**Results:**

Measurements were successful in 38 participants. Twenty-one and seventeen patients were allocated into saline and rocuronium respectively. The number of breaths achieving plateaued capnogram did not differ between the saline (95% C.I.: 6.2 to 12.8 breaths) and rocuronium groups (95% C.I.: 5.6 to 12.7 breaths) (*p* = 0.779). Mean tidal volume changes from breath 1 was significantly greater in rocuronium group than saline group (95% C.I.: 0.56 to 0.99 versus 3.51 to 4.53 ml kg-IBW^−1^, *p* = 0.006). Significantly more patients in rocuronium group (94%) achieved tidal volume greater than 5 mg kg-ideal body weight^−1^ within one minute than those in saline group (62%) (*p* = 0.026). Presence of obstructive sleep apnea did not affect effectiveness of rocuronium for improvement of tidal volume during one-handed mask ventilation.

**Conclusions:**

Use of rocuronium facilitates tidal volume improvement during one-handed mask ventilation even in patients with moderate to severe obstructive sleep apnea.

**Trial registration:**

The clinical trial was registered at (05/12/2013, UMIN000012495): https://upload.umin.ac.jp/cgi-open-bin/ctr_e/ctr_view.cgi?recptno=R000014515

## Background

Anesthesia drugs profoundly depress pharyngeal airway motor neurons and narrows or closes the pharyngeal airway [1–3]. Administration of neuromuscular blockade after loss of consciousness during anesthesia induction may not further deteriorate pharyngeal airway patency. In fact, previous clinical studies indicate no reduction or increase of tidal volume during mask ventilation [4–8] and no impairment or improvement of mask ventilation difficulty [9–11] after the use of depolarizing and non-depolarizing neuromuscular blockades during anesthesia induction.

We previously reported no change of tidal volume after administration of rocuronium without airway maneuvers such as sniffing head posture and triple airway maneuvers in anaesthetized adults with normal airways [5]. A randomized controlled study by Min et al. tested effects of rocuronium on tidal volume during mask ventilation in patients with normal airways and evidenced tidal volume increase in response to earlier administration of rocuronium [7]. Sato et al. demonstrated lower tidal volume during mask ventilation in patients with moderate to severe obstructive sleep apnea [12]. Soltész et al. demonstrated improvement of mask ventilation during progression of muscle paralysis in patients with predictors for difficult face mask ventilation [8]. No previous study has blinded the test drug (rocuronium) by using the placebo drug (saline) to test effectiveness of rocuronium on mask ventilation. A clinical question whether efficiency of one-handed mask ventilation is improved by administration of a non-depolarizing neuromuscular blockade in patients including obstructive sleep apnea remains to be solved.

Accordingly, in this double-blinded randomized placebo-controlled study, we aimed to test a hypothesis that muscle paralysis during anesthesia induction improves one-handed mask ventilation with airway supporting maneuvers in adult patients including moderate to severe obstructive sleep apnea. Changes of capnogram waveform (primary variable) and tidal volume were compared between patients receiving either true (rocuronium) or placebo-drug (saline).

## Methods

### Ethical approval of the study protocol

Ethical approval for this study (Ethical Committee number: 1650) was provided by the Ethical Committee of Chiba University Graduate School of Medicine, Chiba, Japan (Chairperson: Prof. Masaomi Iyo) on 25/11/2013 and revised on July 2017. The study protocol was registered in University hospital Medical information network Clinical trial Registry (UMIN000012495, 05/12/2013: https://upload.umin.ac.jp/cgi-open-bin/ctr_e/ctr_view.cgi?recptno=R000014515). This prospective, double-blinded, and randomized placebo-controlled study with parallel groups from December 2013 to March 2014, and from November 2017 to February 2019 was performed at Chiba University Hospital, Chiba, Japan. Since the study was not completed as originally planned due to absence of the primary investigator (A.I.) at Chiba University Hospital for 3 years, it was restarted after the interruption. All the patients who participated in this study voluntarily signed written informed consent forms after the aim and potential risks of the study were fully explained to each. This study complied with the Declaration of Helsinki and adhered to the applicable CONSORT guidelines.

### Participants

**Fig. 1:**
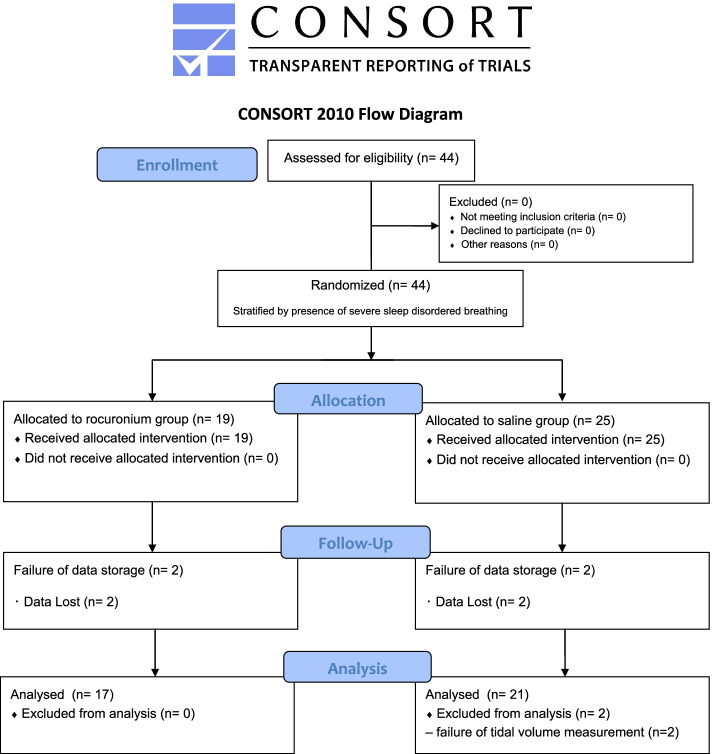
CONSORT diagram of this randomized controlled study

Adult patients (20 to 80 years old) undergoing scheduled surgeries under elective general anesthesia were enrolled. The exclusion criteria were patients who are eligible for awake intubation, patients with full stomach, complete denture, facial malformation, patients with severe comorbidities (American Society of Anesthesiologists Physical Status, more than 2), and patients with allergies to rocuronium or propofol. We approached 44 patients and confirmed their eligibility in this study. All of them agreed to participating on this study. Four participants’ data stored in a computer were lost before analysis (2 participants assigned to rocuronium group and to saline group each). Two patients assigned to saline group were excluded from the analysis due to technical failure of tidal volume measurements (Fig. 1).

### Randomization and allocation

In accordance with the predetermined random allocation tables for patients with and without moderate to severe obstructive sleep apnea defined as apnea hypopnea index (AHI) greater than 20 h^−1^ separately, participants were randomly assigned either to the rocuronium group or to the saline group by the primary investigator (A.I.). The result of randomization was communicated to the investigators (A.I., S.S., K.S.) who injected the drugs, but not to the participant and the anesthesia provider who performed mask ventilation during anesthesia induction (double-blind). Twenty-four anesthesia providers with a wide range of clinical experience of anesthesia management as anesthesia residents or specialists participated in this study.

### Preoperative assessment of sleep-disordered breathing

In addition to preoperative airway assessments, a preoperative assessment of sleep-disordered breathing was performed by a nocturnal portable monitor which measured respiratory airflow and SpO_2_ (SAS2100, Nihon Kohden, Tokyo, Japan). All subjects were instructed to attach an oximetry finger probe and nasal cannula before sleep and to remove them on awakening. After checking quality of the recordings, respiratory and oximetry variables were calculated by computer software. Severity of obstructive sleep apnea was quantified by AHI (moderate obstructive sleep apnea: 15≦AHI < 30 h^−1^, severe obstructive sleep apnea: AHI≧30 h^−1^). The result of the sleep study was informed to the anesthesia provider.

### Anesthesia induction technique and respiratory measurements

Each subject was placed in a supine position and in sniffing head position in the operating room. In addition to pulse oximetry and an electrocardiogram, respiratory variables such as respiratory flow (accuracy: ± 0.025L/s), tidal volume (accuracy: ± 10 ml), airway pressure (accuracy: ± 1cmH_2_O) (GF-220R Multigas/Flow unit, Nihon Kohden, Tokyo, Japan), and CO_2_ concentration with a mainstream capnometer (CAP-ONE, TG-970P, Nihon Kohden, Tokyo, Japan, accuracy: ± 2 mmHg) were continuously monitored. Neuromuscular function with acceleromyography (TOF-Watch; Organon Ireland Ltd., Dublin, Ireland) was assessed by a train of four stimulations every 15 s (no calibration). All these variables were displayed on the cardiorespiratory monitoring screen (Life Scope J, BSM-9100, Nihon Kohden, Tokyo, Japan) and the moving images were captured and stored in a computer for later analyses. In order to blind order of the test drugs to the anesthesia provider who performed mask ventilation, the train-of-four ratio on the display and the forearm with acceleromyography were concealed by drapes.

Two unlabeled but numbered disposable syringes (#1 and #2) containing rocuronium 1.0 mg kg^−1^ (10 mg ml^−1^) and normal saline 0.1 ml kg^−1^ real bodyweight were prepared by one of the investigators (A.I., S.S., K.S.). Both syringes were indistinguishable each other containing liquid with the same volume and clear color. Information of the patient assignment and test drugs was not given to the anesthesia provider who performed mask ventilation and only known to the investigator who injected the test drugs. General anesthesia was induced by intravenous administration of fentanyl 2 μg kg^−1^ and propofol 1 mg kg^−1^ after 3-min inhalation of pure oxygen through an anesthesia circuit. Appropriate anesthesia depth was clinically assessed by the anesthesia provider and additional propofol was injected if necessary. After confirming the loss of consciousness and starting intermittent train of four stimulations through an ulnar nerve by a 50-mA current, the test drug labeled #1 was injected. Twenty seconds after injection of the test drug #1, pressure-controlled ventilation by using an anesthesia machine ventilator through the full-face mask with peak inspiratory pressure 15 cmH_2_O, positive end expiratory pressure of 3 cmH_2_O, and respiratory rate of 15 cycles per minute was started. The anesthesia provider was instructed to perform his/her own best airway opening techniques during one-handed mask ventilation with an anesthesia full-face mask (Air Cushion Face Mask KM202; Koo Medical equipment Co., ltd., China) and a 4-point head strap to prevent air-leakage. Following one-handed mask ventilation for one minute, measurements were continued under two-handed mask ventilation. After approval by the investigator who knew the test drugs, the drapes over the display of train-of-four ratio value and the forearm with acceleromyography were removed and the test drug #2 was injected. Respiratory measurements were performed immediately after tracheal intubation with the same ventilator setting.

### Primary and secondary outcomes

Using the digitized cardiorespiratory monitoring images, effectiveness of mask ventilation was assessed by capnogram waveform and tidal volume. In accordance with 2014 JSA airway guideline which strongly recommends use of capnogram waveform to assess adequacy of mask ventilation [13], we determined the breath number needed to achieve plateaued-capnogram (priorly defined primary variable: needed breaths for plateaued-capnogram) during mask ventilation following #1 test drug injection. As secondary variables, the tidal volume change during mask ventilation particularly focusing on the breath achieving adequate tidal volume defined as normalized tidal volume greater than 5 ml kg-ideal body weight (IBW)^−1^, and level of muscle paralysis were assessed in addition to patient background characteristics.

### Sample size

As characteristics of the primary variable were not previously reported, we used the needed breaths for tidal volume > 5 ml kg-IBW^−1^ for sample size calculation. Mean ± standard deviation of needed breaths for tidal volume > 5 ml kg-IBW^−1^in our previous study were 5.3 ± 5.2 breaths [12]. We expected the similar distribution and 5 breath difference between the groups since we expected the neuromuscular blockade to be effective by 5th breaths. Assuming α = 0.05 (two tailed), β = 0.8, the sample size was determined as 19 patients for each group (SigmaPlot 12.0; Systat Software Inc., Point Richmond, CA). This agreed with our previous result demonstrating statistical significant difference in subgroup analysis of 40 patients [12]. Accordingly, we set the total sample size as 40 patients for this randomized controlled study.

### Statistics

For the baseline variables, summary statistics were constructed using frequencies and proportions for categorical data, and median and IQRs for continuous variables. Breath-by-breath changes of mask ventilation performance was assessed by a mixed effects model for repeated measures (MMRM) with fixed effects for time, group and their interaction, and using an unstructured covariance matrix. The Kenward-Roger method will be used to estimate the degrees of freedom for the fixed effects. These group comparisons were planned. Using background variables listed in Table 1 including age, sex, body mass index, ASA-PS, airway assessment variables, obstructive sleep apnea (no = 1, yes = 2), and anesthesia experience of the anesthesia providers as possible independent explanatory variables, the backward model selection was used for the multiple linear regression analysis. A value of *p* < 0.05 was considered statistically significant, and all p-values were two sided. Statistical analyses were performed using SAS Ver. 9.4 (SAS Institute, Cary, North Carolina, USA) and SigmaPlot 12.0 (Systat Software Inc., Point Richmond, CA).

**Table 1:** Patients’ background variables and anesthesia providers’ characteristics for each of saline and rocuronium groups

	Saline group	Rocuronium group
Number of subjects	21	17
Gender (male (%), female (%))	(11 (52), 10 (48))	(12 (71), 5 (29)
Age (years)	59 (45,71)	58 (52, 68)
Height (m)	1.64 (1.54, 1.69)	1.66 (1.54, 1.72)
Weight (kg)	61.8 (56.2, 71.6)	62.2 (54.5, 78.5)
Body mass index (kg m^−2^)	23.0 (21.5, 26.3)	23.4 (21.5, 28.2)
Neck circumference (cm)	37.5 (34.0, 40.8)	39.0 (35.5, 42.0)
Mallampati class (1 (%), 2 (%), 3 (%), 4 (%))	(10 (48), 7 (33), 3 (14), 1 (5))	(7 (41), 7 (41), 2 (12), 1 (6))
Inter-incisor distance (mm)	46 (40, 50)	50 (42.5, 50.0)
Thyromental distance (mm)	70 (70, 80)	70 (60, 80)
Upper lip bite test (1 (%), 2 (%), 3 (%))	(21 (100), 0 (0), 0 (0))	(12 (71), 5 (29), 0 (0))
ASA Physical Status (1 (%), 2 (%))	(6 (29), 15 (71))	(2 (12), 15 (88))
Apnoea hypopnoea index (hour^−1^)	4.6 (2.0, 22.9)	20.5 (1.5, 37.7)
Number of patients with moderate obstructive sleep apnea: n (%)	5 (24)	5 (29)
Number of patients with severe obstructive sleep apnea: n (%)	2 (10)	5 (29)
Anesthesia providers’ experience (years)	1 (1, 6)	6 (1, 9)
Number of providers with uncertified anesthesiologists: n (%)	12 (57)	6 (35)
Number of certified anesthesiologists: n (%)	9 (43)	11 (65)

Values are median (IQR) or proportion (n (%)). *AHI* Apnea Hypopnea Index, moderate obstructive sleep apnea: 15≦AHI < 30 h^−1^, severe obstructive sleep apnea: AHI≧30 h^−1^

## Results

As shown by a CONSORT flow diagram (Fig. 1), both capnography and tidal volume recordings were successfully performed in 21 patients in saline group and 17 patients in rocuronium group without harm. Although responses to train of four stimulation was not measured in a patient in saline group and 2 patients in rocuronium group, these 3 patients’ data were used for testing the hypothesis and other analyses.

### Results for primary hypothesis: group difference of mask ventilation performance

**Fig. 2:**
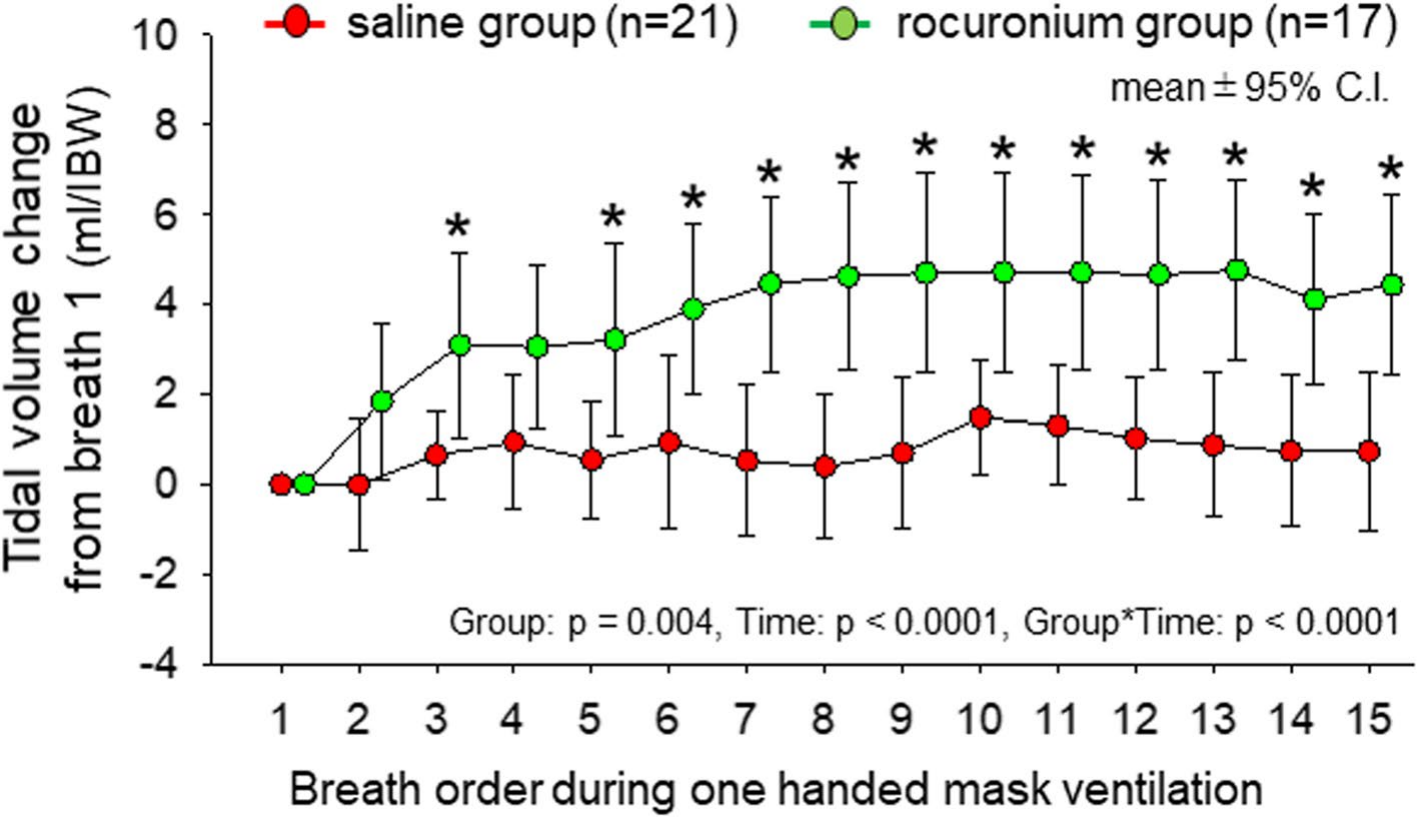
Time course of tidal volume change from breath 1 during 15 cycle one-handed mask ventilation in saline (*n* = 21) and rocuronium (*n* = 17) groups. Symbols and bars represent means and 95% C.I.. Results of a mixed effects model for repeated measures (MMRM) are shown in the figure. * indicates statistically difference between groups (*p* < 0.05)

In rocuronium group patients, train of four ratio at the initiation of mask ventilation was 66 ± 49% and rain of four count at the end of one minute test period was 0.3 ± 0.6. Disappearance of muscular responses to train of four stimulations was achieved at 10.5 (4.5, 15.3) breath in rocuronium group. Table 2 presents differences of mask ventilation performance between the groups. The needed breaths to achieve plateaued-capnogram (primary variable) did not differ between saline and rocuronium groups (95% C.I.: 6.2 to 12.8 versus 5.6 to 12.7 breaths, *p* = 0.779). While the needed breaths to achieve tidal volume greater than 5 mg kg-IBW^−1^ did not differ between the groups (*p* = 0.608), significantly more patients in rocuronium group (*n* = 16, 94%) achieved tidal volume greater than 5 mg kg-IBW^−1^ within one minute than those in saline group (*n* = 13, 62%) (*p* = 0.026). Five patients in saline group had tidal volume less than 2 kg-IBW^−1^ throughout the one-minute measurement period whereas no patient did so in rocuronium group (*p* = 0.053). Mean tidal volume changes from breath 1 was significantly greater in rocuronium group than saline group (95% C.I.: 0.56 to 0.99 versus 3.51 to 4.53 ml kg-IBW^−1^, *p* = 0.006) as illustrated in Fig. 2. While the mean tidal volume did not increase in one minute in saline group, it progressively increased by 7^th^ breath and plateaued afterward in rocuronium group. MMRM indicates significant effects of group (*p* = 0.004) and time (*p* < 0.001) on changes of the mean tidal volume and their significant interaction (*p* < 0.001). There were no significant differences of number of patients achieving tidal volume greater than 5 mg kg-IBW^−1^ with two-handed mask ventilation (0.672) and under tracheal intubation (0.577). Notably, four of eight (50%) saline group patients and one of two (50%) rocuronium group patients who failed to achieve tidal volume greater than 5 mg kg-IBW^−1^ with one-handed mask ventilation also failed to achieve tidal volume greater than 5 mg kg-IBW^−1^ despite application of two-handed mask ventilation. It was successful after tracheal intubation in all of these patients. Although three patients failed to achieve tidal volume greater than 5 mg kg-IBW^−1^ despite tracheal intubation, they achieved it with either one-handed and/or two-handed mask ventilation indicating possible inappropriate ventilator setting for them.

**Table 2:** Differences of mask ventilation performance between saline and rocuronium groups

	saline group (*n* = 21)	rocuronium group (*n* = 17)	*P* value
Needed breaths for plateaued-capnogram	14 (1, 16)	8 (1.5, 16)	0.779
Number of patients achieving plateaued-capnogram in one minute: n (%)	11 (52%)	9 (53%)	1
Needed breaths for TV > 5 ml kg-IBW^−1^	4 (1, 15)	2 (1, 8)	0.608
Number of patients achieving TV > 5 ml kg-IBW^−1^ in one minute: n (%)	13 (62%)	16 (94%)	0.026
Nean TV change from breath 1 during one handed MV (ml kg-IBW^−1^)	0.74 (0.53, 0.94)	4.44 (3.1, 4.7)	0.006
TV at 1st breath during one handed MV (ml kg-IBW^−1^)	3.2 (0, 10.6)	3.4 (0, 6.6)	0.493
TV at 15th breath during one handed MV (ml kg-IBW^−1^)	5.7 (1.6, 9.0)	8.1 (5.1, 11.5)	0.177
TV during two-handed MV (ml kg-IBW^−1^)	7.7 (5.4, 11.1)	9.7 (7.8, 12.4)	0.15
Number of patients achieving TV > 5 ml kg-IBW^−1^ with two-handed MV: n (%)	17 (81%)	15 (88%)	0.672
TV through tracheal tube (ml kg-IBW^−1^)	9.3 (6.5, 10.3)	8.9 (6.2, 9.8)	0.378
Number of patients achieving TV > 5 ml kg-IBW^−1^ under tracheal intubation: n (%)	20 (95%)	15 (88%)	0.577

Values are median (IQR) or proportion (n (%)). Group comparison was performed by Wilcoxon rank sum test

*IBW* Ideal Body Weight, *MV* Mask Ventilation, *TV* Tidal Volume

### Difference of mask ventilation performance between patients with and without obstructive sleep apnea

**Fig. 3:**
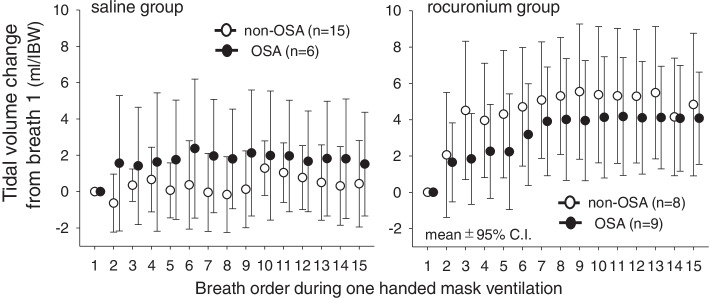
Time course of tidal volume change from breath 1 during 15 cycle one-handed mask ventilation in saline (left pane) and rocuronium (right pane) groups. Open and closed circles represent non-obstructive sleep apnea (non-OSA) and obstructive sleep apnea (OSA) patients, respectively. Symbols and bars represent means and 95% C.I.. A mixed effects model for repeated measures (MMRM) for saline group indicated obstructive sleep apnea: *p* = 0.295, time: *p* = 0.544, and interaction between obstructive sleep apnea and time: *p* = 0.901. MMRM for rocuronium group indicated obstructive sleep apnea: *p* = 0.457, time: *p* < 0.0001, and interaction between obstructive sleep apnea and time: *p* = 0.707

Figure 3 presents differences of mean tidal volume change during one-handed mask ventilation between non-obstructive sleep apnea and obstructive sleep apnea patients in saline (left panel) and rocuronium (right panel) groups. MMRM indicated no difference of mean tidal volume changes between non-obstructive sleep apnea and obstructive sleep apnea patients in both groups (*p* = 0.295, *p* = 0.457, respectively). Notably, no time effect was evident in saline group (*p* = 0.543) while it was clear in rocuronium group (*p* < 0.0001). These indicate beneficial effects of rocuronium on tidal volume increase during mask ventilation regardless of the presence or absence of obstructive sleep apnea.

### Post-hoc analysis: Independent explanatory variables for tidal volume during mask ventilation

Among the background variables, no explanatory variable was determined for the needed breaths achieving plateaued-capnogram (Table 3). Smaller inter-incisor distance and higher AHI were determined as independent predictors for delayed establishment of tidal volume greater than 5 ml kg-IBW^−1^. Higher class of upper lip bite test and thicker neck circumference were determined to be independent predictors for greater improvement of tidal volume during mask ventilation.

**Table 3:** Results of Backwards stepwise regression for explaining tidal volume during mask ventilation

Dependent variable	Independent explanatory variables	Estimate	Standardized partial regression coefficients	Standard error	*P* value
Needed breaths for plateaued-capnogram	No explanatory variable				
Needed breaths for TV > 5 ml kg-IBW^−1^	Inter-incisor distance	-0.329	-0.399	0.129	0.015
	Apnea hypopnea index	0.134	0.347	0.0607	0.033
Mean TV change from breath 1	Upper lip bite test	3.45	0.326	1.567	0.034
	Neck circumference	0.309	0.367	0.125	0.018

*IBW* Ideal Body Weight, *TV* Tidal Volume

## Discussion

In this randomized placebo-controlled study, we found that in adult patients immediately after induction of general anesthesia, 1) rocuronium administration did not contribute to earlier establishment of plateaued capnogram during one-handed mask ventilation compared with saline administration, 2) rocuronium administration resulted in greater increase in tidal volume than saline administration during one-handed mask ventilation, and 3) rocuronium administration was equally effective to both patients with and without obstructive sleep apnea for tidal volume improvement during one-handed mask ventilation.

There are several methodological considerations to properly interpret results of this study. Firstly, the primary variable, number of breaths needing to achieve plateaued-capnogram, was predetermined because of the recommendation of capnogram usage for assessing adequacy of mask ventilation in JSA airway guideline [13], but it appeared not to be sensitive to detect the timing of establishment of adequate mask ventilation compared with tidal volume measurements. Furthermore, the predetermined sample size was based on the tidal volume greater than 5 ml kg-IBW^−1^ as adequate mask ventilation performance and effect size turned to be smaller than expected. Accordingly, we consider the primary hypothesis should be alternatively assessed by using tidal volume parameters instead of capnogram waveform as many previous studies successfully identified significant improvement of tidal volume after administration of muscle blockades [4–8]. In fact, we successfully detected significant group differences of tidal volume parameters such as number of patients achieving tidal volume greater than 5 ml kg-IBW^−1^ in one minute and mean tidal volume change from breath 1 as shown in Table 2. Secondly, the interpretation of the results of this study is limited due to uneven distribution of gender and obstructive sleep apnea patients between the groups while the same randomization and allocation principal was used throughout the study period. However, since difficulty of mask ventilation is expected in severe obstructive sleep apnea patients, the primary hypothesis would have been supported if more patients with severe obstructive sleep apnea were to be included in the saline group. Thirdly, the timing of initiation of mask ventilation might have significantly influenced the results of this study according to the finding by Min et al. [7, 14]. Level of muscle paralysis at twenty seconds after injection of rocuronium adopted in this study might not have been sufficient to produce the meaningful group difference of muscle paralysis. We consider this is unlikely because the adductor pollicis responses to train of four stimulations to ulnar nerve was 66 ± 49% in rocuronium group. Previous animal and human studies demonstrated more rapid onset of muscle paralysis in the masseter muscles, which paralysis may be essential for performance of airway opening maneuvers by the anesthesiologists, and higher sensitivity to non-depolarizing muscle blockage compared with the adductor pollicis [15–17]. Fourthly, we did not control the mask ventilation technique except use of one hand and use of the head strap. Efficacy of one-handed mask ventilation would be influenced by the sealing technique as well as the airway opening techniques. The use of the head strap is not our routine, but commonly chosen by the anesthesiologists particularly when the leakage management is difficult with one hand. We asked to use the head strap to measure tidal volume accurately by preventing the leakage for the purpose of this study. The pressure produced by the head strap on the mandible would have negatively influenced on effectiveness of the airway opening technique. Fifthly, we only assessed effect of rocuronium on mask ventilation during anesthesia induction and did not assess incidence of critical airway events such as severe hypoxemia and associated cardio-respiratory complications during and after surgery. Clinical values of rocuronium administration should be determined by overall clinical impact of the drug. Lastly, our study was not designed to test the hypothesis in patients with difficulty of performing airway maneuvers due to severely-limited mandible advancement and/or head extension in whom two-handed mask ventilation immediately after anesthesia induction or awake intubation is usually indicated [13, 18–20]. Future studies may need to test effectiveness of neuromuscular blockade on two-handed mask ventilation in these high-risk patients in order to achieve safe and comfortable anesthesia induction for all surgical patients.

In this study, a constant driving pressure to ventilate the lung was maintained by anesthesia ventilator with a fixed pressure-controlled ventilation mode under use of properly-fitting anesthesia facemask with a head strap. The changes of mask ventilation should reflect changes of pharyngeal airway patency in response to airway maneuvers by anesthesia provider. This is also supported by our previous finding that rocuronium did not change tidal volume during mask ventilation without airway maneuvers [5]. Accordingly, we consider that more forward movement of the mandible or greater neck extension during the triple airway maneuvers to be the major mechanism for progressive improvement of mask ventilation during progression of muscle paralysis after rocuronium administration.

We found severity of obstructive sleep apnea (higher AHI) was an independent risk factor for delayed establishment of normal tidal volume during mask ventilation. However, patients with possible small mandible (higher class of upper lip bite test) and those with thicker neck, common features of obstructive sleep apnea patients [21], showed significant improvement in tidal volume during mask ventilation. Although our post-hoc analysis indicates equal effectiveness of rocuronium administration for tidal volume improvement during one-handed mask ventilation in severe obstructive sleep apnea patients, this does not guarantee maintenance of normal tidal volume during one-handed mask ventilation and safety of airway management since pharyngeal patency in obstructive sleep apnea before application of airway maneuvers must be significantly impaired than non-obstructive sleep apnea patients. Triple airway maneuvers should be properly applied in obstructive sleep apnea patients. Although use of muscle relaxant may facilitate mandibular advancement and neck extension, oral ventilation route should be secured by mouth opening as smaller inter-incisor distance was an independent risk factor for delayed establishment of normal tidal volume during one-handed mask ventilation in accordance with previous studies [22, 23].

## Conclusions

Use of rocuronium facilitates improvement of tidal volume during one-handed mask ventilation even in patients with moderate to severe obstructive sleep apnea.

## Data Availability

The datasets used and/or analysed during the current study are available from the corresponding author on reasonable request.

## Abbreviations

AHI: Apnea hypopnea index
ASA-PS: American Society of Anesthesiologists Physical Status
IBW: Ideal body weight
IQR: Interquartile range
JSA: Japanese Society of Anesthesiologists
MMRM: Mixed effects model for repeated measures
SpO_2_: Arterial oxygen saturation
TV: Tidal volume
